# Effect of a Home‐Base Core Stability Exercises in Hereditary Ataxia. A Randomized Controlled Trial. A Pilot Randomized Controlled Trial

**DOI:** 10.1002/mdc3.14036

**Published:** 2024-04-02

**Authors:** Rosa Cabanas‐Valdés, Helena Fernández‐Lago, Selma Peláez‐Hervás, Laura Serra‐Rusiñol, Carlos López‐de‐Celis, Maria Masbernat‐Almenara

**Affiliations:** ^1^ Department of Physiotherapy, Faculty of Medicine and Health Sciences Universitat Internacional de Catalunya Barcelona Spain; ^2^ Department of Nursing and Physiotherapy Universitat de Lleida Lleida Spain; ^3^ Research group of health care. IRB Lleida, Institute for Biomedical Research Dr. Pifarré Foundation Lleida Spain; ^4^ Group on Society Studies, Health, Education and Cures, University of Lleida Lleida Spain; ^5^ Servicio de Rehabilitación, Hospital Clínic de Barcelona Barcelona Spain; ^6^ Neurosalud Barcelona Barcelona Spain; ^7^ Fundació Institut Universitari per a la recerca a l'Atenció Primària de Salut Jordi Gol i Gurina (IDIAP Jordi Gol) Barcelona Spain

**Keywords:** hereditary ataxias, cerebellar ataxia, core stability exercises, balance, trunk function, quality of life

## Abstract

**Background:**

Core stability exercises (CSE) have been shown to be effective in improving trunk function in several neurological diseases, but the evidence is scarce on Hereditary Ataxias (HA).

**Objective:**

To evaluate the effectiveness of a 5‐week home‐based CSE program in terms of ataxia severity, trunk function, balance confidence, gait speed, lower limb motor function, quality of life, health status and falls rate in HA individuals at short‐ and long‐term.

**Methods:**

This is an assessor‐blind randomized controlled clinical trial parallel group 1:1. The individuals were divided in experimental group (EG) performed standard care in addition to CSE, and control group (CG) performed standard care alone. The CSE home‐program was conducted 1‐h/day, 5‐day/week for 5‐week. The assessment was performed at baseline, endpoint (5‐week), and follow‐up (10‐week). The primary outcomes were ataxia severity assessed by the Scale for the Assessment and Rating of Ataxia and trunk function assessed by Spanish‐version of Trunk Impairment Scale 2.0. The secondary outcomes were balance confidence assessed by Activities‐specific Balance Confidence (ABC), gait speed by 4‐meter walk test (4‐MWT), the lower limb motor function by 30‐s sit‐to‐stand, quality of life by EuroQol 5‐dimension 5‐level (EQ‐5D‐5L), health‐status by EQ‐5D and falls rate.

**Results:**

Twenty‐three HA individuals were recruited (51.8 ± 11.10 years). Statistically significant group‐time interaction was shown in ABC (F:5.539; *P* = 0.007), EQ‐5D‐5L Total (F:4.836; *P* = 0.013), EQ 5D (F:7.207; *P* = 0.006).

**Conclusions:**

No statistical differences between groups for ataxia severity and trunk function were observed. However, were differences for balance confidence, gait speed, quality of life, and falls rate in HA individuals.

Hereditary ataxias (HA) are a clinically and genetically heterogeneous group of degenerative diseases of the cerebellum, brainstem, and spinal cord.^1^ Although the cerebellum is the unifying site of pathology in inherited cerebellar ataxias, the clinical phenotype differs between ataxias. HA are subdivided into the autosomal‐recessive ataxias as Friedreich's ataxia,^2^ the autosomal‐dominant ataxias designated as spinocerebellar ataxias,^3^ and X‐linked ataxias with fragile X‐associated tremor ataxia syndrome. The autosomal recessive is estimated to affect 3.3 per 100,000 and autosomal dominant 2.7 per 100,000 people worldwide.^4^

Ataxia is usually caused by cerebellar dysfunction or an altered vestibular or proprioceptive afferent input to the cerebellum.^5^ Postural disorders in HA individuals^6^ causes imbalance, gait impairments and falls.^7^ They have deficiencies in trunk local stability^8^ and trunk position sense compared with age‐matched healthy individuals.^9^ Trunk endurance plays a critical role in maintaining optimal postural control.^10^ The core stability is the ability to control the position and movement of the trunk over the pelvis to allow optimal production, transfer and control of power towards the limbs.^11^ The goal of core stability exercises (CSE) is to improve and recover the ability to control the spine and hip in order to promote neuromuscular control, coordination, strength and resistance of the muscles of the lumbopelvic region.^12^ CSE is a keystone in many areas of neurological rehabilitation such as Parkinson's disease,^13^ multiple sclerosis,^14^ and stroke.^15^, ^16^, ^17^ CSE training is recommended for HA individuals by Sheppard et al.^18^ To the best of our knowledge there are not studies that assessed a home‐based CSE program in HA population. There are only two studies that evaluated CSE on‐site. One^19^ showed significant improvement in balance and another in dynamic trunk balance in HA children.^20^

It is important to note that HA individuals must train consistently due to the fact that long‐term benefits depend on a frequent training.^21^ It is essential to encourage this population to involve and increase the self‐efficacy, engagement and self‐responsibility in the recovery process.^21^ However, they perceived barriers to exercise as lack of time, family dependents, financial constraints, convenience and forgetting.^22^

Providing a home‐based exercise program to individuals is one of the most important aspects of physiotherapy.^23^ A home‐based programme^24^ in addition to a face‐to‐face personalized treatment could be beneficial to improve their symptoms.^25^ In addition, it can also provide a mean to optimize the use of healthcare and financial resources in front of a progressive, often function‐limiting, degenerative HA disease.^26^ There is some evidence to suggest HA population do well with home exercise programmes.^27^, ^28^ However, for many people with HA benefits from more individual therapy are not sustained with a home program. To tolerate long‐term engagement, prescribed exercises should be enjoyable, meaningful, satisfying and appropriately challenging.^25^

Therefore, the primary purpose of this study was to determine whether a home‐based CSE program in addition to standard care improves ataxia severity and trunk function in HA individuals. The secondary outcomes were to improve balance confidence, gait speed, lower limb motor function, quality of life, health status and falls rate.

## Methods

This is an assessor‐blind randomized controlled clinical trial parallel group 1:1. CONSORT guidelines were followed. The study was carried out as per the standards set by the Declaration of Helsinki 2013 and it was registered on ClinicalTrials.gov number: NCT04750850.

### Participants

The inclusion criteria were adults over 18 years old suffering a diagnosed HA, as spinocerebellar ataxia, Friedreich's ataxia, idiopathic sporadic cerebellar ataxia, and specific neurodegenerative disorders in which ataxia is the dominant symptom. They must be able to understand and execute simple instructions. As exclusion criteria were concurrent neurologic disorder or major orthopedic problem that hamper sitting balance, relevant psychiatric disorders that may prevent from following instructions and other treatments that could influence the effects of the interventions, any severe or moderate contraindication to practice physical activity and suffering from a musculoskeletal impairment that prevents him/her from performing the exercises. All participants signed an informed consent before being included into the study. Information on patients' age and the type of HA were collected from medical records. The participants were recruited on an outpatient basis from Catalan association of HA and *Hospital Clínic Barcelona* from May 2021 to December 2022. First, a webinar was held on the benefits of CSE for this population. It was organized by the Catalan association of HA and interested participants later contacted the principal researcher.

Participants of both groups, the experimental group (EG) and control group (CG) performed their standard care. It could include physiotherapy or/and physical activity (cycling or walking or swimming) and medication. The exercise intensity depended on the ataxia severity. Physiotherapy emphasized improvement of postural balance with balance training including turning, dynamic balance exercise in standing and walking, range‐of‐motion exercises for trunk and limbs, muscle strengthening, and up and down stairs.

**Figure 1:**
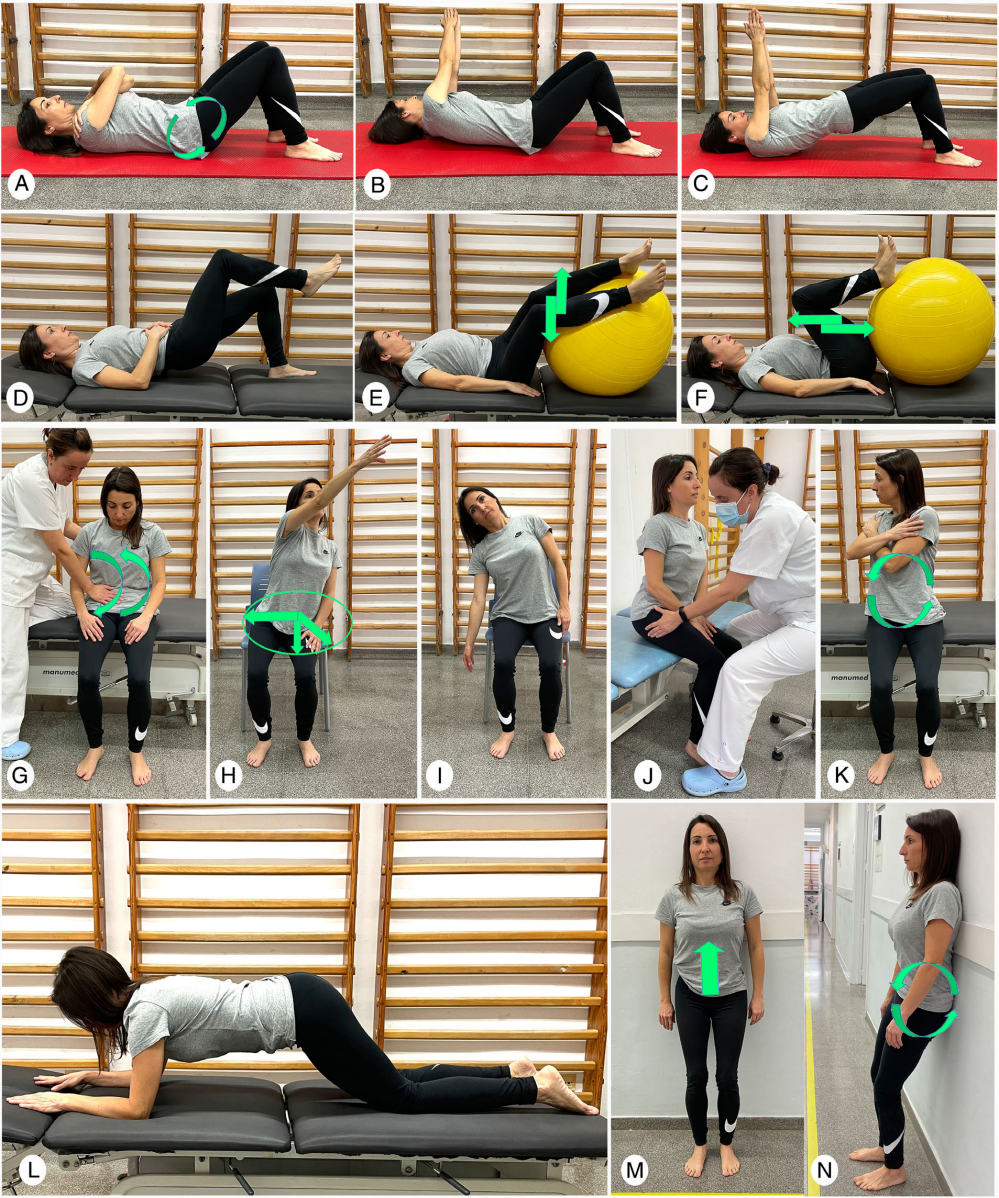
Core stability exercises program: (**A**) Lumbopelvic anteversion + retroversion (**B)** Upper trunk rotation (**C**) Bridging (**D**) Unilateral bridging (**E**) Lower limbs rotation (**F**) Knee‐hip flexion‐extension (**G**) Trunk flexion‐extension (**H**) Reaching in three directions (forward, ipsilateral, contralateral) (**I**) Trunk lateral inclination (**J**) Lift hemipelvis (**K**) Upper trunk rotation, (**L**) Modified Plank (**M**, **N**) Lumbopelvic anteversion + retroversion.

The EG performed a home‐based CSE program in addition to their standard care. This CSE program was initially designed for stroke survivors,^29^ but it was adapted for HA population (Fig. 1). The principal investigator and three physiotherapists (HF, SP, LS) worked on the adaptation of this program. These physiotherapists have a master's degree in Neurorehabilitation and more than 5 years of experience with neurological individuals. For the adaptation of the program, the characteristics of the HA individuals were considered, as they have mainly ataxia, postural and proprioception impairments. For this reason, the patients were instructed to use both arms with their elbows extended. This allowed them to move their trunk more easily. The number of exercises were also reduced. A standing and a modified plank exercises were introduced. The seated exercises on a physioball were limited to only those had the possibility to perform them safely. It is focused on strengthening the muscles of the trunk (intrinsic/extrinsic), as well as improving proprioception. CSE program was performed in different positions, supine position, sitting on a stable and unstable surface or prone position. They were instructed in the use of the 5 points of the Borg 10 Rating of Perceived Exertion^29^ for self‐monitoring exercise effort intensity. A physiotherapist (LS) made an initial home visit to train each patient individually the correct execution of the CSE program. It was performed twice/day in 30‐min sessions held 5‐day/week for 5‐week at the individual's home. The intensity and increase in difficulty of the program was tailored to the patient depending on their disabilities to perform exercises and progress to more challenging exercises. Once the exercises were learnt and done correctly by the patient, he/she was instructed to do it alone by himself/herself alongside a booklet with photographs and instructions of the personalized CSE program, and if required, the help of his caregiver or family member. Once a week, the physiotherapist telephoned or made a videoconference to the patient in case they had any doubts and encouraged them to do the exercises. If necessary, the physiotherapist could go back to the patient's home to evaluate the correct execution of the CSE.

The CG continued with their standard care. For ethical reasons, once the study was completed, the participants assigned to the CG were given the opportunity to receive the same 5‐week home‐based CSE program after 10 weeks.

### Outcome Measures

The principal researcher instructed an assessor (SP). The participants were assessed blindly at baseline, at 5‐week, and at 10‐week. Once these 10 weeks were over, the participants of CG could perform CSE and were assessed at the end of 5‐week.

Ataxia severity was assessed through the Scale for the Assessment and Rating of Ataxia (SARA).^30^ This scale has an excellent test–retest reliability (correlation coefficient = 0.90), inter‐rater reliability (correlation coefficient = 0.98), and internal consistency was high as indicated by Cronbach's alpha = 0.94.^31^ It includes eight items related to gait (0–8 points), stance (0–6 points), sitting (0–4 points), speech disturbance (0–6 points), finger‐chase test, nose‐finger test, fast alternating movements and heel‐shin test (0–4 points). The total score ranges from 0 (no ataxia) to 40 (most severe ataxia). The motor assessment of the four limbs (items 5–8) is performed bilaterally, and the mean values are used to obtain the total score.

Trunk function was assessed by the Spanish‐version of the Trunk Impairment Scale 2.0 (S‐TIS 2.0).^32^ It is a reliable and validated scale to measure trunk impairments and trunk performance in ambulatory patients with neuromuscular diseases.^33^ The highest possible total score is 16 points with two subscales (dynamic sitting‐balance and coordination). If the patient cannot hold it without support of the back and arms, with the hands on the thighs, the feet in contact with the ground, and the knees bent at 90° for 10‐s, the score total is 0 points.

The balance confidence was assessed by the Activities‐specific Balance Confidence (ABC).^34^ It is a structured questionnaire (16 questions) that measures the confidence of an individual during activities of daily living. ABC demonstrates excellent internal consistency and has data to assist in measuring changes in individuals with stable neurologic conditions and their reliability has been assessed.^35^ Its use is recommended by a clinical practice guideline^35^ and Ataxia Global Initiative working group.^36^ ABC is correlated with gait variability,^37^ shows a strong correlation with lateral velocity change, which was used to quantify dynamic balance during turning, and it is an important aspect of daily living.^36^

Gait speed was assessed by the 4‐meters walk test^38^ (4‐MWT) meters/second (m/s). It has an excellent test–retest reliability, concurrent validity, and strong correlation between 4‐MWT and 10 Meter Walk test with a dynamic start at comfortable speed. To perform the 4‐MWT is necessary a 6‐meter walkway to provide two acceleration/deceleration zones of 1‐meter, marked with an adhesive tape and two cones. Participants started with their toes behind the starting line. They walked at their preferred gait speed three times, and them were averaged.

The lower limb muscle motor function was assessed by the 30‐s sit‐to‐stand test.^39^ It has a good construct validity as a measure of lower extremity function in people with multiple sclerosis.^40^ It is performed on a chair without armrest; the participant is seated with the arms crossed and held against the chest and must perform as many full stands as possible within 30 s.

Quality of life was assessed by EuroQol 5 dimensions 5 levels (EQ‐5D‐5L).^41^ Its suitability as an outcome measure for treatment studies in HA population that aim to ameliorate ataxia or slow down disease progression remains to be determined.^42^ It has a 5‐component scale including mobility, self‐care, usual activities, pain/discomfort, and anxiety/depression from 0 (best possible) to 5 points (worst possible). Health status was evaluated by EQ‐5D, which is a visual analogue scale for health ranging from 0 (worst possible) to 100 (best possible).

Participants were asked to document the CSE completed in an exercise diary. The patient's adherence to the CSE program was assessed by the ratio of attendance (calculated by dividing the number of sessions attended by the number of scheduled sessions). Falls rate (people who have experienced falls) and adverse events were also recorded during the 10‐week period.

### Sample Size

The calculation was performed for a bilateral contrast, assuming an alpha risk of 0.05 and a beta risk of 0.1 with GRANMO (v.7.12). We estimated a typical standard deviation of 0.7 points based on the study by Miyai et al.^43^ We were assuming a difference of 1 point (SARA) as minimum clinically important difference according to the study of Barbuto et al.^28^ We estimated a 5% loss to follow‐up to be necessary. Therefore, 11 individuals per group were needed.

Participants were randomized via Random.org web‐based randomization system 1:1 into two groups: EG (home‐based CSE program plus standard care); or CG (standard care). The generated sequence was hidden in opaque envelopes by the principal investigator.

### Statistical Analysis

It was performed by a person outside the study. The characterization of the sample was based on a descriptive statistical analysis, presented through frequencies and percentages. The mean value and standard deviation were calculated for quantitative variables in both groups. SPSS 26.0 was used to assess group differences in all variables at each time interval. Descriptive analysis was carried out calculating mean and standard deviation for quantitative variables, or median and interquartile ranges. The Shapiro–Wilk test was used to determine non‐normal distribution of quantitative data. A repeated‐measure analysis of variance (ANOVA) with time and group was conducted to determine changes in the outcomes. A Chi‐square test was performed for falls rate. Effect size (ES) was calculated using eta squared (ŋ^2^).^44^ An effect size >0.14 was considered as large, around 0.06 as medium, and <0.01 small. If the assumption of sphericity was violated, the Greenhouse–Geisser correction was utilized for interpretation. When a statistically significant interaction effect was noted, a post‐hoc analysis was performed, and the Bonferroni correction was used to adjust for multiple comparisons. In the comparison between groups, the differences between T1 and T0 and T2–T0 were calculated, and a one‐way ANOVA was performed. Statistical analysis was performed by intention‐to‐treat. The significance level was set at *p* < 0.05.

## Results

**Figure 2:**
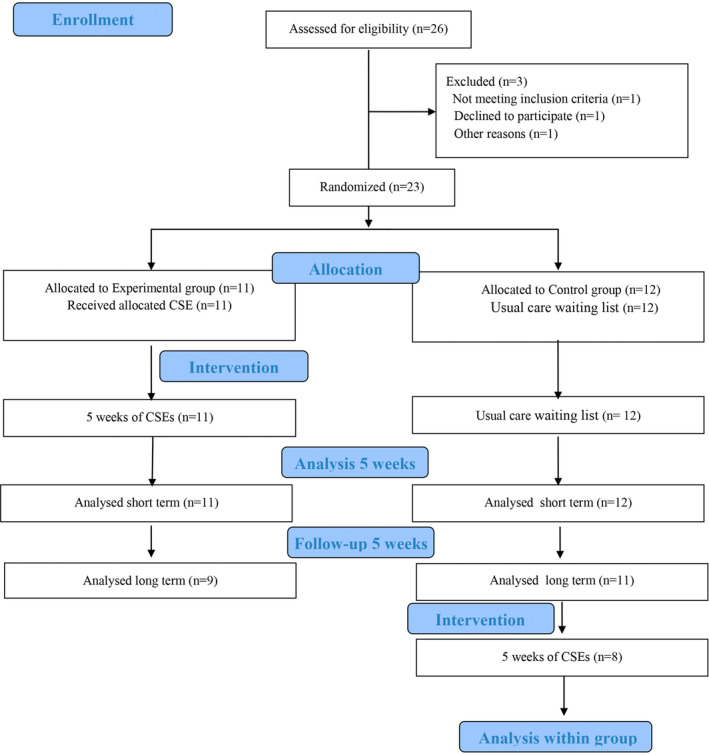
Flow chart of the study.

Twenty‐six patients were selected for trial screening and a total of 23 individuals (mean age = 51.8 ± 11.10) (Fig. 2). One individual did not meet the inclusion criteria, other declined to participate and other became very sick. They were divided in two groups: EG (11) and CG (12). After the end of the follow‐up phase, 8 HA individuals of CG performed home‐based CSE program. Drop‐outs were because one individual moved house and lived far away, so could not be assessed, another became very ill and was admitted to hospital, another was admitted to a nursing home and refused to participate, and another was given the exercises on paper but did not do them because he had depression.

Demographic variables were recorded at baseline (Table 1). No significant difference was shown between both groups for any of the variables. Regarding adherence, four individuals were able to perform the CSE program 2 times/day (30‐min session) during 5‐weeks. Five individuals needed help from a caregiver to perform the CSE program. One patient suffered from back pain for 14 days (but was able to continue doing the CSE in decubitus supine position). Those who walked only indoors conducted the CSE in supine, prone and sitting position on a chair or bed. No individuals reported falls while performing the CSE program.

**TABLE 1:** Characteristics of the participants at baseline

	Experimental Group (n:11)	Control Group (n:12)
Sex *n* (%)		
Male, *n* (%)	6 (54.5%)	5 (58.3%)
Female, *n* (%)	5 (45.5%)	7 (41.7%)
Age (years), mean (SD)	53.2 ± 13.8	50.8 ± 8.7
Weight (Kg), mean (SD)	67.9 ± 12.8	61.6 ± 10.3
High (cm), mean (SD)	167.8 ± 0.8	167.6 ± 0.9
Body mass index	24.0 ± 3.3	21.9 ± 2.8
Physical therapy of standard care, n (%)	6 (54.5%)	12 (100%)
Outdoor ambulant, n (%)	11 (100%)	8 (66.6%)
Physical therapy time/week mean (SD)	1 (3) *	1.6 (2.8) *
Physical activity of standard care, n (%)	9 (81.8%)	8 (66.7%)
Physical activity of standard care h/week	3.9 ± 2.8	3 (6.5) *
Falls (people)		
Yes *n* (%)	9 (81.8%)	10 (83.3%)
No *n* (%)	2 (9.2%)	2 (6.7%)
Disease duration, years	77.9 ± 50.4	93.3 ± 37.4
Falls rate, mean (SD)	2.1 ± (2.3)	2.5 (2.8)
Type of ataxia		
SCA 1	1 (8.3%)	4 (36.4%)
SCA 3	6 (50.0%)	3 (27.3%)
SCA 4	1 (8.3%)	‐
SCA 7	‐	1 (9.1%)
SCA 18	‐	1 (9.1%)
CANVAS	1 (8.3%)	‐
Friedreich ataxia	3 (25.0%)	2 (18.2%)

Abbreviations: CANVAS, cerebellar ataxia with neuropathy and bilateral vestibular areflexia syndrome; SCA, Spinocerebellar ataxia.

* Median (IQR).

Table S1 shows the data and within‐group and between groups differences at 5 and 10 weeks. Statistically significant group‐time interaction was only showed in the balance confidence ABC (F:5.539; *P* < 0.007; df:2), quality of life EQ‐5D‐5L Total (F:4.836; *P* < 0.013;df:2), health status EQ‐5D (F:7.207; *P* < 0.006, df:1.46), and 4‐MWT (F:6.818; *P* = 0.007; df:1.45). In ABC variable regarding within‐group analysis, the EG showed a statistically significant difference at 10 weeks with the Bonferroni correction (*P* = 0.044). A statistically significant difference in ABC variable was also revealed between groups analysis at 5 weeks (F:6.067; *P* = 0.022) and 10 weeks (F:7.612; *P* = 0.012). Concerning to the EQ‐5D‐5L total score EG showed a statistically significant difference at 10 weeks with the Bonferroni correction (*P* = 0.033) in the within‐group analysis. A statistically significant difference was also indicated between groups at 5 weeks (F:5.892; *P* = 0.024) and 10 weeks (F:6.887; *P* = 0.016). The EQ‐5D did not show statistically significant difference in the within‐group analysis. Even so, a statistically significant difference was revealed between groups at 5 weeks (F:5.047; *P* = 0.038) and 10 weeks (F:9.002; *P* = 0.007). The 4‐MWT, showed a statistically significant difference at 5 weeks with the Bonferroni correction (*P* = 0.002) in the within‐group analysis, and statistically significant difference, was also revealed between groups analysis at 5 weeks (F:13.024; *P* = 0.002).

The supplementary tables show the data at 5 and 10 weeks in the subscales of S‐TIS 2.0 (Table S2), sections of EQ‐5D‐5L (Table S3) and sections of SARA (Table S4). Additionally, we observed in the analysis of the subscales EQ‐5D‐5L mobility (F:4.915, *P* = 0.012, df:2), self‐care (F:3.926, *P* = 0.042, df:1.46), and usual activities (F:3.527, *P* = 0.038, df:2), presented an interaction between group‐time. None showed significant within‐group statistical differences. The EQ‐5D‐5L mobility subscale showed a statistically significant differences between groups at 5 weeks (*P* = 0.034) and 10 weeks (*P* = 0.017). The self‐care showed a statistically significant difference between groups at 5 weeks (*P* = 0.036), and the usual activities demonstrated a statistically significant difference between groups at 10 weeks (*P* = 0.028) (Table S3).

According to the falls rate (people who have experienced falls) there was no difference at baseline between groups the CG (83.3%) and the EG (81.8%) *P* = 0.924. At 5‐week, the CG reduced to 50.0% and the EG to 18.2%, with no statistically significant difference (*P* = 0.145). However, at 10‐weeks, the CG increased to 66.7%, while the EG remained at 18.2%, this difference being statistically significant (*P* = 0.019). The adherence rate was 75%.

## Discussion

The results of this study showed differences between groups favoring the EG (5‐week home‐based CSE program in addition to standard care) in terms of balance confidence, quality of life (mobility, self‐care, usual activities), health status, gait speed and falls rate in HA individuals. These results were maintained at follow‐up except for gait speed. Concerning ataxia severity, trunk function and lower limb strength motor function were shown statistical differences within‐group in the EG.

Regarding ataxia severity, we expected a change in SARA total score, equal to or greater than 1 point between groups. However, no differences between groups were observed. Nevertheless, a difference within‐group was observed for the EG (−1.64 points). It is in line with two studies^20^, ^45^ that performed supervised CSE in children and balance training, respectively. Compared to prior studies with home‐based balance exercises training our result was higher than Barbuto et al.^28^ (−0.60 points balance group) and Keller et al.^27^ (no differences). In other studies^43^, ^46^, ^47^ that performed supervised balance training, improvements in ataxia severity were initially observed. However, when they performed home‐based balance exercises then ataxia returned to its baseline state.

Concerning trunk function assessed by S‐TIS 2.0 no difference between‐group was shown. However, a difference EG within‐group analysis revealed (1 point). It is according to Yigit et al.^20^ with TIS. Nonetheless, they showed improvements for dynamic trunk balance with Modified Functional Reach test.

In relation to balance confidence measured by the ABC scale a difference between groups were observed favoring the EG at short‐term and long‐term. In addition, when CG performed the CSE (after 10‐week), they also improved by 3.98 points at the end of the program. Surprisingly, no differences were shown by Keller et al.^27^ Our results for the SARA gait/stance score were better in EG than in CG. It should be considered that in balance rehabilitation, confidence building is as important as physical training and it is correlated with balance performance.^48^ Tabbassum et al.^19^ observed differences between groups for dynamic balance.

According to our results, the CSE provided an improvement in the EQ‐5D‐5L (mobility, self‐care, usual‐activities, health‐status) at short‐ long‐term. Despite the fact that the variable quality of life is important for this population,^49^ no studies were focused on it. Regarding health status assessed by EQ‐5D our results were positive at short‐ long‐term for EG. They were in accordance with Milne et al.^46^

We used patient‐reported outcome measures (PROMs) to assess balance confidence, quality of life and health status. The use of PROMs is essential in an individual level as part of a personalized care and it has been considered of great added value, as it may provide insight into patients’ perceived health and their needs.^50^

Relative to comfortable walking speed was observed at 0.82 ± 0.38 m/s in EG. This is an important result since gait speed has been shown to reflect overall functional independence and good quality of life.^51^ Our results are like Keller et al.^27^ (0.9 ± 0.3 m/s), by Barbuto et al.^28^ balance group (0.89 ± 0.30 m/s) and by Miyai et al.^43^ (0.95 ± 0.071 m/s).

A decrease in the number of falls was observed for EG at 10‐week (18.2%) and a statistically significant difference was observed *P* = 0.019. This fact is in line with Tabbassum et al.^19^ where they observed an improvement for falls score within the EG group in HA.

The cerebellum is an essential motor and cognitive structure of the nervous system^52^ and it has an important role in motor learning.^53^ Home‐based CSE program has a role to play, and the benefits HA individuals received are positive. Our results have a translation to real‐world and have some advantages/disadvantages. Firstly, the participants were supervised by a physiotherapist only the first day of the intervention and the rest of the time they were autonomous. Secondly, the session was divided in two times day to avoid fatigue. Thirdly, the CSE intervention evolved from being performed firstly in a supine, prone position and progressed to seated and standing as the patient's confidence increased. This can be beneficial to the non‐ambulant outdoor individuals. In contrast, our patients were unsupervised, which meant that some patients did not dare to sitting exercises on a physioball for fear of falling.

Tabbassum et al.^19^ performed a CSE program in addition to balance training. Intervention was performed in a sequential motor learning through skill learning that includes coordination of the deep and superficial muscles of the trunk in different activities as turning, walking sideways, heel to toe walking, sit to stand, heel lifts, toe lifts, single leg standing, leg swings, stair climbing. Yigit et al.^20^ executed a CSE program in addition to functional trunk training in HA children. Comparisons interventions protocols between these different studies and ours are made difficult because CSE is combined with its standard care. The other studies^44^, ^46^, ^47^ mentioned above consisted in balance training in different positions such as sitting, standing, sit‐to‐stand, and walking sideways, heel to toe walking, heel lifts, and stair climbing. These program were always monitored by a physiotherapist. Therefore, individuals could exercise safely on unstable surfaces.

### Limitations

This study has several limitations. Both groups received their standard care, which was uncontrolled therapeutic approach. As a pilot study, and one of the first to systematically evaluate a CSE home‐program intervention in HA, we considered it essential not to add complexity by introducing an active comparator in CG, which should be included in future studies. Although we did the sample calculation, the sample is small. Of importance is the lack of objective assessments of motor improvements after intervention, such as posturography and biomechanical analyses. Individuals were not blinded to CSE, and this may influence the results of the PROMs, however the primary outcomes were objective.

In summary, no differences between groups were observed for ataxia severity and trunk function. However, a 5‐week home‐based CSE program is promising to treat balance confidence, quality of life and falls rate for HA individuals. Thus, our results stimulate further studies of home‐based CSE programs as an element of rehabilitation in HA population.

## Author Roles

(1) Research project: A. Conception, B. Organization, C. Execution; (2) Statistical Analysis: A. Design, B. Execution, C. Review and Critique; (3) Manuscript: A. Writing of the first draft, B. Review and Critique.

R.C.‐V.: 1A, 1B, 2C, 3B.

C.L.‐C.: 2A, 2B.

H.F.‐L.: 1C, 3B.

S.P.‐H.: 1C, 3B.

L.S.‐R.: 1C.

M.M.‐A.: 1C, 3A, 3B.

## Disclosures

**Ethical Compliance Statement:** The study protocol was approved by the ethical committee from *Universitat Internacional de Catalunya* and *Hospital Clinic de Barcelona* with number FIS‐2021‐03 and HCB/2022/0436 respectively. Informed consent was obtained of all patients. We confirm that we have read the Journal's position on issues involved in ethical publication and affirm that this work is consistent with those guidelines.

**Funding Sources and Conflict of Interest:** College of Fisioterapeutes of Catalunya, number: R01/20 and the authors declare that there are no conflicts of interest relevant to this work.

**Financial Disclosures for the Previous 12 Months:** The authors declare that there are no additional disclosures to report.

## Supporting information


**TABLE S1.** Outcomes measures within‐group and between‐groups comparisons total scores at short‐ and long‐term.

**TABLE S2.** Outcomes measures within‐group and between‐groups comparisons for S‐TIS 2.0 subscales at short‐ and long‐term.

**TABLE S3.** Outcomes measures within‐group and between‐groups comparisons for EQ‐5D‐5L sections at short‐ and long‐term.

**TABLE S4.** Outcomes measures within‐group and between‐groups comparisons for SARA sections at short‐ and long‐term.
