# Supplementary material for: Effect of a Home‐Base Core Stability Exercises in Hereditary Ataxia. A Randomized Controlled Trial. A Pilot Randomized Controlled Trial
Source: Mov Disord Clin Pract. 2024 Apr 2;11(6):666–75. doi: 10.1002/mdc3.14036 (PMC11145153; doi:10.1002/mdc3.14036)
Supplement: Supplementary file 1 — TABLE S1. Outcomes measures within‐group and between‐groups comparisons total scores at short‐ and long‐term. [file MDC3-11-666-s002.docx]

| **STable 1**: Outcomes measures within-group and between-groups comparisons total scores at short- and long-term | | | | | | | | | | | | | | |  |  |
| --- | --- | --- | --- | --- | --- | --- | --- | --- | --- | --- | --- | --- | --- | --- | --- | --- |
|  | T0 | T1 | Difference T1-T0 | | | | | | | T2 | Difference T2-T0 | | | | | |
|  | Mean ± SD | Mean ± SD | Mean | 95% CI | within group  *p* | | ES | Between groups  *p* & ES | | Mean ± SD | Mean | 95% CI | within  group  *p* | ES | Between groups  *p* & ES | |
| ABC Balance |  |  |  |  |  | |  |  | |  |  |  |  |  |  | |
| Experimental Group | 53.05 ± 24.70 | 56.73 ± 25.55 | 3.68 | [-1.003; 8.359] | 0.161 | | 0.01 | ***p*=0.022**  ŋ^2^=0.21 | | 58.69 ± 24.38 | 5.64 | [0.118; 11.155] | **0.044** | 0.01 | *p*=**0.012**  ŋ^2^=0.25 | |
| Control Group | 35.07 ± 30.27 | 32.61 ± 29.03 | -2.46 | [-6.940; 2.024] | 0.318 | | 0.00 |  |  | 32.61 ± 29.03 | -2.48 | [-7.750; 2.817] | 0.714 | 0.00 |  |  |
| S-TIS 2.0 Total |  |  |  |  |  | |  |  | |  |  |  |  |  |  | |
| Experimental Group | 9.36 ± 1.86 | 10.36 ± 2.77 | 1.00 | [-0.380; 2.380] | 0.220 | | 0.04 | *p*=0.503  ŋ^2^=0.02 | | 9.72 ± 3.32 | 0.36 | [-2.121; 1.394] | 1.000 | 0.00 | *p*=0.638  ŋ^2^=0.01 | |
| Control Group | 7.58 ± 4.06 | 8.08 ± 4.54 | 0.50 | [-0.821; 1.821] | 0.508 | | 0.00 |  |  | 7.67 ± 5.00 | 0.08 | [-1.600; 1.766] | 1.000 | 0.00 |  |  |
| Sit-to-Stand 30 seconds |  |  |  |  |  | |  |  | |  |  |  |  |  |  | |
| Experimental Group | 7.82 ± 5.17 | 8.55 ± 5.45 | 0.73 | [-0.619; 2.073] | 0.523 | | 0.01 | *p*=0.443  ŋ^2^=0.03 | | 8.73 ± 5.82 | 0.91 | [-0.575; 2.393] | 0.378 | 0.01 | *p*=0.413  ŋ^2^=0.03 | |
| Control Group | 5.08 ± 5.60 | 5.25 ± 5.80 | 0.17 | [-1.122; 1.455] | 1.000 | | 0.00 |  |  | 5.33 ± 5.93 | 0.25 | [-1.171; 1.671] | 1.000 | 0.00 |  |  |
| EQ-5D-5L Total |  |  |  |  |  | |  |  | |  |  |  |  |  |  | |
| Experimental Group | 10.36 ± 2.94 | 9.45 ± 2.91 | -0.91 | [-1.871; 0.053] | 0.068 | | 0.02 | *p*=**0.024**  ŋ^2^=0.20 | | 9.00 ± 2.28 | -1.36 | [-2.638; -0.089] | **0.033** | 0.06 | *p*=**0.016**  ŋ^2^=0.023 | |
| Control Group | 13.67 ± 4.75 | 14.00 ± 4.51 | 0.33 | [-0.587; 1.254] | 1.000 | | 0.00 |  |  | 14.08 ± 4.48 | 0.42 | [-0.804; 1.637] | 1.000 | 0.00 |  |  |
| EQ-5D Health status |  |  |  |  |  | |  |  | |  |  |  |  |  |  | |
| Experimental Group | 67.73 ± 19.41 | 70.45 ± 16.80 | 2.73 | [-1.071; 6.525] | 0.227 | | 0.01 | *p*=**0.038**  ŋ2=17 | | 71.36 ± 16.60 | 3.64 | [-1.720; 8.993] | 0.276 | 0.01 | *p*=**0.007**  ŋ^2^=0.028 | |
| Control Group | 55.50 ± 23.71 | 53.75 ± 23.94 | -1.75 | [-5.386; 1.886] | 0.673 | | 0.00 |  |  | 50.58 ± 23.90 | -4.92 | [-10.045; 0.212] | 0.063 | 0.01 |  |  |
| 4-MWT (meters/second) |  |  |  |  |  | |  |  | |  |  |  |  |  |  | |
| Experimental Group | 0.69 ± 0.41 | 0.82 ± 0.38 | 0.14 | [0.050 ;0.225] | **0.002** | | 0.03 | p=0.002  ŋ2=0.38 | | 0.76 ± 0.36 | 0.07 | [-0.038;0.179] | 0.312 | 0.01 | *p*=0.056  ŋ^2^=0.016 | |
| Control Group | 0.56 ± 0.53 | 0.53 ± 0.51 | -0.03 | [-0.115;0.053] | 1.000 | | 0.00 |  |  | 0.52 ± 0.52 | -0.05 | [-0.150;0.058] | 0.793 | 0.00 |  |  |
| SARA Total ataxia severity | |  |  |  | |  |  | |  |  |  |  |  |  |  | |
| Experimental Group | 11.91 ± 7.37 | 10.27 ± 7.64 | -1.64 | [-3.305; 0.032] | 0.056 | | 0.01 | *p*=0.215  ŋ^2^=0.07 | | 10.13 ± 7.97 | -1.77 | [-3.651; -0.106] | 0.069 | 0.01 | *p*=0.607  ŋ^2^=0.011 | |
| Control Group | 17.25 ± 11.59 | 16.75 ± 10.90 | -0.50 | [-2.098; 1.098] | 1.000 | | 0.00 |  |  | 16.00 ± 10.26 | -1.25 | [-3.048; 0.548] | 0.255 | 0.00 |  |  |
| ABC: Activities-specific Balance Confidence, EQ-5D-5L: EuroQol 5 dimensions 5 levels, SARA: Scale for the Assessment and Rating of Ataxia, S-TIS 2.0: Spanish-version of Trunk Impairment Scale 2.0, 4-MWT: 4-meter walk test, T0: baseline, T1: post-treatment, T2: follow-up, ES: effect size. | | | | | | | | | | | | | | | | |
