# Supplementary material for: Effect of a Home‐Base Core Stability Exercises in Hereditary Ataxia. A Randomized Controlled Trial. A Pilot Randomized Controlled Trial
Source: Mov Disord Clin Pract. 2024 Apr 2;11(6):666–75. doi: 10.1002/mdc3.14036 (PMC11145153; doi:10.1002/mdc3.14036)
Supplement: Supplementary file 2 — TABLE S2. Outcomes measures within‐group and between‐groups comparisons for S‐TIS 2.0 subscales at short‐ and long‐term. [file MDC3-11-666-s004.docx]

| **STable 2**: Outcomes measures within-group and between-groups comparisons for S-TIS 2.0 subscales at short- and long-term | | | | | | | | | | | | |  |
| --- | --- | --- | --- | --- | --- | --- | --- | --- | --- | --- | --- | --- | --- |
|  | T0 | T1 | Difference T1-T0 | | | | | T2 | Difference T2-T0 | | | | |
|  | Mean ± SD | Mean ± SD | Mean | 95% CI | within  group  *p* | ES | Between groups  *p* & ES | Mean ± SD | Mean | 95% CI | within  group  *p* | ES | Between groups  *p* & ES |
| S-TIS 2.0 Dynamic sitting balance | |  |  |  |  |  |  |  |  |  |  |  |  |
| Experimental Group | 7.00 ± 1.73 | 7.73 ± 1.79 | 0.73 | [-0.227; 1.682] | 0.182 | 0.04 | *p*=0.547  ŋ^2^=0.02 | 6.82 ± 2.44 | -0.18 | [-1.565; 1.201] | 1.000 | 0.00 | *p*=0.984  ŋ^2^=0.00 |
| Control Group | 5.58 ± 2.97 | 6.00 ± 3.13 | 0.42 | [-0.497; 1.331] | 0.526 | 0.00 |  | 5.42 ± 3.40 | -0.17 | [-1.491; 1.158] | 1.000 | 0.00 |  |
| S-TIS 2.0 Coordination |  |  |  |  |  |  |  |  |  |  |  |  |  |
| Experimental Group | 2.36 ± 1.03 | 2.64 ± 1.57 | 0.27 | [-0.475; 1.021] | 1.000 | 0.01 | *p*=0.503  ŋ^2^=0.02 | 2.18 ± 1.17 | -0.18 | [-0.983; 0.619] | 1.000 | 0.01 | *p*=0.322  ŋ^2^=0.04 |
| Control Group | 2.00 ± 1.60 | 2.08 ± 1.56 | 0.08 | [-0.633; 0.799] | 1.000 | 0.00 |  | 2.25 ± 1.76 | 0.25 | [-0.517; 1.017] | 1.000 | 0.01 |  |
| S-TIS 2.0: Spanish-version of Trunk Impairment Scale 2.0, T0: baseline; T1: post-treatment; T2: follow-up, ES: effect size. | | | | | | | | | | | | | |
