# Supplementary material for: Effect of a Home‐Base Core Stability Exercises in Hereditary Ataxia. A Randomized Controlled Trial. A Pilot Randomized Controlled Trial
Source: Mov Disord Clin Pract. 2024 Apr 2;11(6):666–75. doi: 10.1002/mdc3.14036 (PMC11145153; doi:10.1002/mdc3.14036)
Supplement: Supplementary file 3 — TABLE S3. Outcomes measures within‐group and between‐groups comparisons for EQ‐5D‐5L sections at short‐ and long‐term. [file MDC3-11-666-s003.docx]

| **STable 3**: Outcomes measures within-group and between-groups comparisons for EQ-5D-5L sections at short- and long-term | | | | | | | | | | | | |  |
| --- | --- | --- | --- | --- | --- | --- | --- | --- | --- | --- | --- | --- | --- |
|  | T0 | T1 | Difference T1-T0 | | | | | T2 | Difference T2-T0 | | | | |
|  | Mean ± SD | Mean ± SD | Mean | 95% CI | within  group  *p* | ES | Between groups  *p* & ES | Mean ± SD | Mean | 95% CI | within  group  *p* | ES | Between  groups  *p* & ES |
| EQ-5D-5L Mobility |  |  |  |  |  |  |  |  |  |  |  |  |  |
| Experimental Group | 3.09 ± 0.94 | 2.82 ± 0.98 | -0.27 | [-0.707; 0.161] | 0.351 | 0.02 | *p*=**0.034**  ŋ^2^=0.18 | 2.73 ± 0.79 | -0.36 | [-0.810; 0.083] | 0.134 | 0.04 | *p*=**0.017**  ŋ^2^=0.23 |
| Control Group | 3.33 ± 1.30 | 3.58 ± 1.31 | 0.25 | [-0.166; 0.666] | 0.573 | 0.01 |  | 3.58 ± 1.24 | 0.25 | [-0.177; 0.677] | 0.428 | 0.01 |  |
| EQ-5D-5L Self-care |  |  |  |  |  |  |  |  |  |  |  |  |  |
| Experimental Group | 1.91 ± 0.94 | 1.73 ± 0.90 | -0.18 | [-0.682; 0.319] | 1.000 | 0.01 | *p*=**0.036**  ŋ^2^=0.19 | 1.64 ± 0.81 | -0.27 | [-0.740; 0.224] | 0.504 | 0.02 | *p*=0.061  ŋ^2^=0.15 |
| Control Group | 2.50 ± 1.38 | 2.92 ± 1.24 | 0.42 | [-0.063; .896] | 0.103 | 0.03 |  | 2.75 ± 1.36 | 0.25 | [-0.226; 0.726] | 0.183 | 0.01 |  |
| EQ-5D-5L Usual activities | |  |  |  |  |  |  |  |  |  |  |  |  |
| Experimental Group | 2.45 ± 1.04 | 2.18 ± 1.08 | -0.27 | [-0.623; 0.077] | 0.167 | 0.02 | *p*=0.158  ŋ^2^=0.07 | 2.00 ± 0.89 | -0.45 | [-0.950; -0.041] | 0.080 | 0.05 | *p*=**0.028**  ŋ^2^=0.20 |
| Control Group | 3.08 ± 1.16 | 3.08 ± 1.08 | 0.00 | [-0.335; 0.335] | 1.000 | 0.00 |  | 3.25 ± 0.97 | 0.17 | [-0.308; 0.641] | 1.000 | 0.01 |  |
| EQ-5D-5L Pain |  |  |  |  |  |  |  |  |  |  |  |  |  |
| Experimental Group | 1.45 ± 0.52 | 1.27 ± 0.47 | -0.18 | [-0.455; 0.092] | 0.295 | 0.03 | *p*=0.506  ŋ^2^=0.02 | 1.18 ± 0.40 | -0.27 | [-0.698; 0.153] | 0.331 | 0.08 | *p*=0.242  ŋ^2^=0.06 |
| Control Group | 2.33 ± 0.78 | 2.25 ± 0.75 | -0.08 | [-0.345; 0.179] | 1.000 | 0.00 |  | 2.33 ± 0.89 | 0.00 | [-0.407; 0.407] | 1.000 | 0.00 |  |
| EQ-5D-5L Anxiety |  |  |  |  |  |  |  |  |  |  |  |  |  |
| Experimental Group | 1.45 ± 0.69 | 1.45 ± 0.93 | 0.00 | [-0.428; 0.428] | 1.000 | 0.00 | *p*=0.285  ŋ^2^=0.05 | 1.45 ± 0.93 | 0.00 | [-0.492; 0.492] | 1.000 | 0.00 | *p*=0.350  ŋ^2^=0.04 |
| Control Group | 2.42 ± 1.00 | 2.17 ± 0.84 | -0.25 | [-0.660; 0.160] | 0.382 | 0.02 |  | 2.17 ± 0.83 | -0.25 | [-0.721; 0.221] | 1.000 | 0.02 |  |
| EQ-5D-5L: EuroQol 5 dimensions 5 levels, T0: baseline, T1: post-treatment, T2: follow-up, ES: effect size. | | | | | | | | | | | | | |
