# Supplementary material for: Effect of a Home‐Base Core Stability Exercises in Hereditary Ataxia. A Randomized Controlled Trial. A Pilot Randomized Controlled Trial
Source: Mov Disord Clin Pract. 2024 Apr 2;11(6):666–75. doi: 10.1002/mdc3.14036 (PMC11145153; doi:10.1002/mdc3.14036)
Supplement: Supplementary file 4 — TABLE S4. Outcomes measures within‐group and between‐groups comparisons for SARA sections at short‐ and long‐term. [file MDC3-11-666-s001.docx]

| **STable 4**: Outcomes measures within-group and between-groups comparisons for SARA sections at short- and long-term | | | | | | | | | | | | |  |  |
| --- | --- | --- | --- | --- | --- | --- | --- | --- | --- | --- | --- | --- | --- | --- |
|  | T0 | T1 | Difference T1-T0 | | | | | T2 | Difference T2-T0 | | | | | |
|  | Mean ± SD | Mean ± SD | Mean | 95% CI | within group  p | ES | Between groups  p & ES | Mean ± SD | Mean | 95% CI | within group  p | ES | Between groups  p & ES | |
| SARA Gait |  |  |  |  |  |  |  |  |  |  |  |  |  | |
| Experimental Group | 3.64 ± 2.25 | 3.55 ± 2.42 | -0.09 | [-0.679; 0.498] | 1.000 | 0.00 | p=0.981  ŋ^2^=0.07 | 3.36 ± 2.50 | -0.27 | [-0.843; 0.297] | 0.681 | 0.00 | p=0.254  ŋ^2^=0.05 | |
| Control Group | 4.75 ± 2.99 | 4.67 ± 3.03 | -0.08 | [-0.647; 0.480] | 1.000 | 0.00 |  | 4.83 ± 3.01 | 0.08 | [-0.463; 0.629] | 1.000 | 0.00 |  |  |
| SARA Stance |  |  |  |  |  |  |  |  |  |  |  |  |  | |
| Experimental Group | 2.18 ± 1.47 | 2.27 ± 2.10 | 0.09 | [-0.934; 1.115] | 1.000 | 0.00 | p=0.377  ŋ^2^=0.03 | 2.27 ± 2.10 | 0.09 | [-1.068; 1.250] | 1.000 | 0.00 | p=0.377  ŋ^2^=0.03 | |
| Control Group | 2.75 ± 2.53 | 3.33 ± 2.50 | 0.58 | [-0.398; 1.564] | 0.567 | 0.01 |  | 3.33 ± 2.64 | 0.58 | [-0.526; 1.693] | 0.557 | 0.01 |  |  |
| SARA Sitting |  |  |  |  |  |  |  |  |  |  |  |  |  | |
| Experimental Group | 0.91 ±1.64 | 0.55 ± 1.29 | -0.36 | [-1.567; 0.840] | 1.000 | 0.01 | p=0.735  ŋ^2^=0.01 | 0.36 ± 1.21 | -0.55 | [-1.592; 0.501] | 0.569 | 0.03 | p=0.830  ŋ^2^=0.00 | |
| Control Group | 1.58 ± 1.88 | 1.00 ± 1.65 | -0.58 | [-1.736; 0.569] | 0.606 | 0.03 |  | 0.92 ± 1.56 | -0.67 | [-1.669; 0.335] | 0.295 | 0.03 |  |  |
| SARA Speech disturbance | |  |  |  |  |  |  |  |  |  |  |  |  | |
| Experimental Group | 1.09 ± 1.22 | 0.91 ± 1.04 | -0.18 | [-0.713; 0.349] | 1.000 | 0.01 | p=0.527  ŋ^2^=0.02 | 1.18 ± 0.98 | 0.09 | [-0.546; 0.727] | 1.000 | 0.00 | p=0.982  ŋ^2^=0.00 | |
| Control Group | 1.67 ± 1.72 | 1.67 ± 1.30 | 0.00 | [-0.509; 0.509] | 1.000 | 0.00 |  | 1.75 ± 1.54 | 0.08 | [-0.526; 0.693] | 1.000 | 0.00 |  |  |
| SARA Finger chase |  |  |  |  |  |  |  |  |  |  |  |  |  | |
| Experimental Group | 1.18 ± 0.72 | 0.45 ± 0.57 | -0.73 | [-1.325; -0.130] | 0.014 | 0.24 | p=0.483  ŋ^2^=0.02 | 0.50 ± 0.59 | -0.68 | [-1.381; 0.018] | 0.058 | 0.21 | p=0.609  ŋ^2^=0.01 | |
| Control Group | 1.58 ± 1.65 | 1.08 ± 1.24 | -0.50 | [-1.072; 0.072] | 0.101 | 0.03 |  | 0.71 ± 0.75 | -0.88 | [-1.545; -0.203] | 0.008 | 0.10 |  |  |
| SARA Nose-finger |  |  |  |  |  |  |  |  |  |  |  |  |  | |
| Experimental Group | 1.00 ± 0.63 | 0.86 ± 0.45 | -0.14 | [-0.582; 0.309] | 1.000 | 0.02 | p=0.085  ŋ^2^=0.13 | 0.86 ± 0.71 | -0.14 | [-0.629; 0.356] | 1.000 | 0.01 | p=0.786  ŋ^2^=0.13 | |
| Control Group | 1.21 ± 0.92 | 1.50 ± 0.77 | 0.29 | [-0.135; 0.718] | 0.269 | 0.03 |  | 1.00 ±0.60 | -0.21 | [-0.680; 0.263] | 0.790 | 0.01 |  |  |
| SARA Heel-shin slide |  |  |  |  |  |  |  |  |  |  |  |  |  | |
| Experimental Group | 1.14 ±0.78 | 1.05 ± 0.91 | -0.09 | [-0.778; 0.597] | 1.000 | 0.00 | p=0.751  ŋ^2^=0.01 | 1.05 ± 0.85 | -0.09 | [-0.557; 0.375] | 1.000 | 0.00 | p=0.718  ŋ^2^=0.01 | |
| Control Group | 2.04 ± 1.36 | 1.83 ± 1.34 | -0.21 | [-0.867; 0.450] | 1.000 | 0.01 |  | 2.04 ± 1.42 | 0.00 | [-0.446; 0.446] | 1.000 | 0.00 |  |  |
| SARA Fast alternating hand movements | |  |  |  |  |  |  |  |  |  |  |  |  | |
| Experimental Group | 0.77 ± 0.61 | 0.64 ± 0.60 | -0.14 | [-0.637; 0.364] | 1.000 | 0.01 | p=0.614  ŋ^2^=0.01 | 0.55 ± 0.52 | -0.23 | [-0.626; 0.172] | 0.459 | 0.04 | p=0.916  ŋ^2^=0.00 | |
| Control Group | 1.67 ± 1.30 | 1.67 ± 1.13 | 0.00 | [-0.479; 0.479] | 1.000 | 0.00 |  | 1.42 ± 1.22 | -0.25 | [-0.632; 0.132] | 0.310 | 0.01 |  |  |
| SARA: Scale for the Assessment and Rating of Ataxia, T0: baseline, T1: post-treatment, T2: follow-up, ES: effect size. | | | | | | | | | | | | | | |
